# An update on the management of breast cancer in Africa

**DOI:** 10.1186/s13027-017-0124-y

**Published:** 2017-02-14

**Authors:** V. Vanderpuye, S. Grover, N. Hammad, PoojaPrabhakar, H. Simonds, F. Olopade, D. C. Stefan

**Affiliations:** 1 National center for Radiotherapy and Nuclear Medicine, Korle-Bu Teaching Hospital, Accra, Ghana; 2 Hospital of University of Pennsylvania, Department of Radiation Oncology, (Botswana-UPENN program), 3400 Civic Center Blvd., Philadelphia, PA 19104 USA; 3 Cancer Centre of Southeastern Ontario, Burr 2, Kingston General Hospital, 25 King Street W, Kingston, ON K7L 5P9 Canada; 4 University of Texas Southwestern Medical Center, Dallas, TX USA; 5 Division of Radiation Oncology, Tygerberg Hospital/University of Stellenbosch, Tygerberg, South Africa; 6 The University of Chicago, 5841 S Maryland Avenue, MC 2115, Chicago, IL 60637 USA; 7 Walter Sisulu University Nelson Mandela Dr, Nelson Mandela Drive, Mthatha, 5100 Eastern Cape South Africa

**Keywords:** Breast cancer, Radiotherapy, Chemotherapy, Targeted therapies, Survival, Hormonal therapy

## Abstract

**Background:**

There is limited information about the challenges of cancer management and attempts at improving outcomes in Africa. Even though South and North Africa are better resourceds to tackle the burden of breast cancer, similar poor prognostic factors are common to all countries. The five-year overall Survival rate for breast cancer patients does not exceed 60% for any low and middle-income country (LMIC) in Africa. In spite of the gains achieved over the past decade, certain characteristics remain the same such as limited availability of breast conservation therapies, inadequate access to drugs, few oncology specialists and adherence to harmful socio-cultural practices. This review on managing breast cancer in Africa is authored by African oncologists who practice or collaborate in Africa and with hands-on experience with the realities.

**Methods:**

A search was performed via electronic databases from 1999 to 2016. (PubMed/Medline, African Journals Online) for all literature in English or translated into English, covering the terms “breast cancer in Africa and developing countries”. One hundred ninety were deemed appropriate.

**Results:**

Breast tumors are diagnosed at earlier ages and later stages than in highincome countries. There is a higher prevalence of triple-negative cancers. The limitations of poor nursing care and surgery, inadequate access to radiotherapy, poor availability of basic and modern systemic therapies translate into lower survival rate. Positive strides in breast cancer management in Africa include increased adaptation of treatment guidelines, improved pathology services including immuno-histochemistry, expansion and upgrading of radiotherapy equipment across the continent in addition to more research opportunities.

**Conclusion:**

This review is an update of the management of breast cancer in Africa, taking a look at the epidemiology, pathology, management resources, outcomes, research and limitations in Africa from the perspective of oncologists with local experience.

## Background

Publications on breast cancer in Africa start by describing a large number of patients presenting with advanced disease, limited access to cancer education, screening, and care. We have learned from previous studies that registries are still missing in Africa or are only hospital based in most regions of the continent. The estimates of breast cancer incidence are presented as figures but not with the real data as the current situation, remains still to be determined [1].

Survival is seldom described and if so only selectively in a limited number of countries or centers. Cancer mortality rates in African countries are not comparable to those of high-income countries (HIC) [1], reaching unacceptable high proportions.

The Concorde −2 study of 5-year breast cancer survival from 1995 to 2009 based on the analysis of individual data from 279 population-based registries in 67 countries, reported that, in HIC, age-standardized net survival rates were more than 85%. One country in Africa, Mauritius, a HIC island nation off the coast of Madagascar, had similar survival rates of 87.4% (95% CI:78.1–96.7). North African countries had lower outcomes compared to HIC, for example, 59.8% (95% CI:48.6–71.1) in Algeria, 76.6% (95% CI:55.5–97.7) in Libya (Benghazi registry) and 68.4% (95% CI:64.5–72.2) in Tunisia. By contrast, data available from three Sub-Saharan countries, South Africa 53.4% (95% CI:35.5–71.3), The Gambia 11.9%(95% CI:0–24.7) and Mali 13.6%(95% CI:0, 0–30.1), were significantly inferior to other countries around the world [1]. More than 50% of African women diagnosed with breast cancer die of the disease [2]. The disease is the most frequent cause of cancer death in less developed regions, causing one in five deaths in African women [3], described as a new “shift” from the previous decade [4]. Breast tumors are diagnosed a decade or two lower on age at diagnosis and present at advanced stages compared to developed countries [5, 6]. A higher prevalence of hormone receptor negative and triple-negative cancers(TNBC) is found in Africa [7]. The paucity of oncology specialist including nurses and surgeons, access to radiotherapy, availability of basic and modern systemic and hormonal therapies and steadfast adherence to negative socio-cultural beliefs is reflected in the observed lower survival rates compared to high-income countries. In spite of the various setbacks, improvements in breast cancer management include the development or adaptation of treatment guidelines, improved pathology services including immunohistochemistry testing for hormone receptor testing. The expansion and upgrading of radiotherapy resources, new collaborations fostered between international organizations and African cancer treatment facilities will promote research opportunities and improve outcomes.

Previous reviews were specific to surgery, stage at presentation, pathology services or discussed sub-regions whereas this paper provides an update on the current state of breast cancer management in Africa as whole, looking at epidemiology, clinical presentation, and access to radiotherapy, systemic therapies, pathology services, outcomes and new research.

## Methods

A search was performed via electronic databases (Pubmed, Medline, African Journal online) for literature in English or translated into English covering all aspects of breast cancer in Africa from 1999 to 2016. One thousand three hundred and twenty articles on the subject were retrieved under the terms “breast cancer and Africa or developing countries.” Publications with high emphasis on prevention and screening were excluded. Two hundred and twenty-five articles were relevant to the subject under review and One hundred and ninety articles including 15 review articles, were selected.

## Results

The majority of publications were from North and West Africa (60%), others were from East (10%), Central and South Africa (12%). Other publications (31%) broadly discussed breast cancer in larger geographical regions i.e., Sub-Saharan Africa, developing countries in which discussions specific to Africa were highlighted. Publications from authors in developed countries and collaborating international organizations were found in high impact journals whereas publications from local health personnel were found in lower impact journals. There were very few prospective studies and many retrospective studies.

New research findings, epidemiology, practice update, challenges involved in disease control, differences in disease characteristics, management practices, and outcomes are highlighted.

## Epidemiology

Non-communicable diseases, including breast cancer, are on the rise in Africa, presumably due to advances in health care, translating into longer life expectancy and increased detection of cancer. Breast cancer incidence increases with age, and people in Africa are living longer due to better control of human immunodeficiency virus (HIV) and other infectious diseases [8]. This section will discuss prevalence, incidence, and risk factors of breast cancer in Africa. Eleven studies were found on the epidemiology of breast cancer in Africa, including studies from Zimbabwe, Tunisia, Egypt, Morocco, South Africa, and Nigeria.

### Prevalence

Breast cancer is leading cancer among the female population worldwide, as well as in the majority of countries in Africa, according to data from 26 African countries for 2012 [9]. In Africa, breast cancer is responsible for one in four diagnosed cancers and one in five cancer deaths in women [10].

### Incidence

Marked variation exists in the reported incidence of breast cancer worldwide – from 95 to 100 cases per 100,000 persons in North America, Northern Europe, and Australia to 13.5–30 per 100,000 women in sub-Saharan Africa(SSA) [3]. The breast cancer incidence in Africa continues to increase and is projected to double by 2050 [11]. In Zimbabwe, a 4.5% annual increase in breast cancer incidence over the period 1991–2010 has been noted [11]. Within SSA, there is considerable regional variation in the estimated incidence of breast cancer, with 38.9 (per 100,000 women) in Southern Africa, 38.6 in western Africa, 30.4 in eastern Africa, and 26.8 in central Africa [3]. The high rates in southern Africa and urban parts of Africa may be due to better reporting and a higher population of Anglo-Europeans in those areas [12]. Of note, studies from Tunisia, Egypt, and Morocco report that North Africa has a greater proportion of inflammatory breast cancer (IBC) among all breast cancers than elsewhere in the world; however, the incidence of IBC in North Africa is in decline [13–15]. As only a few African countries maintain cancer registries, accurate prevalence figures are unavailable, although the global burden of cancer study (GLOBOCAN) data estimate that in 2012, 94,000 women developed breast cancer [3].

### Risk factors

Several studies have investigated breast cancer risk and various reproductive and anthropometric factors. Use of oral or injectable contraceptives within the previous 10 years significantly increased risk of breast cancer in South Africa, with an odds ratio (OR) of 1.66 and 95% confidence interval (CI) of 1.28–2.16 [16]. Another study in Nigeria found an inverse relationship between age at menarche and breast cancer risk (OR: 0.72; 95% CI: 0.54–0.95) [17]. In a Nigerian study of 1233 breast cancer cases, body mass index (BMI) had an inverse relationship to risk (OR: 1.22; 95% CI: 1.14–1.32) [18]. Also in Nigeria, height, waist circumference (OR: 2.39; 95% CI: 1.59–3.60), and waist-to-hip ratio (OR: 2.15; 95% CI: 1.61–2.85) showed positive correlation to breast cancer [18, 19]. A study for North Africa defines a risk profile in Egyptian and Tunisian women that are more protective about high-income countries. These are a higher mean number of children, younger mean age at first pregnancy, longer mean duration of breastfeeding, lower mean age at menopause, lower prevalence of contraceptive use and lower alcohol consumption [13].

In summary, breast cancer is the most prevalent cancer in African women, although incidence is lower than in high-income countries. Even though the risk factors of breast cancer in Africa are similar to those in high-income countries, the variation in risk factor incidences may account for the differences between African countries and high-income countries.

### Clinical presentation

The clinical presentation of breast cancer in African women is significantly different compared to their counterparts in high-income countries, as well as from Africa.

### Age at presentation

Breast cancer patients in Africa present at a relatively younger age compared to patients in high-income countries [7, 20, 21]. The overall mean age of presentation in West African women is between 35 and 45 years, 10 to 15 years earlier than in women from high-income countries [22]. Similarly, a 3-year retrospective review of 374 breast cancer patients in Kenya showed a median age of 44 years [6], while the mean age in a Tanzanian cancer registry was 44.7 years [23].

### Stage at presentation

The majority of patients in Africa present with advanced stage breast cancer, with 89.6% and 72.8% of breast patients in Kenya and Nigeria respectively presenting with advanced stage disease [6, 24]. These rates are relatively higher than rates of advanced stage breast cancer in high-income countries [25]. Studies in South Africa reported an advanced stage breast cancer incidence of 50 and 55% [25, 26]. However, a Moroccan study reported an incidence of 33% for Stage III and IV breast cancers [27]. Of note, south and northern African patients present at earlier stages compared to the rest of Africa. Table 1. summarizes the data for clinical presentation in the studies reviewed [6, 7, 23–28].

**Table 1:** Clinical presentation of breast cancer by study included in this review

Study	Country	Patients	Reported incidence of advanced stage breast cancer	Median Age at Diagnosis	Receptor Status in HIV- uninfected patients
Otheino-Abinya [6]	Kenya	250	89.6%	44	
Ikpat [28]	Nigeria	300		42.7	
Adebamowo [24]	Nigeria	250	72.8%	43	
Anyanwu [7]	Nigeria	179	72%	46.9	
Cubasch [25]	Soweto, South Africa	1092	50%		ER: 58%
					PR: 47%
					HER2: 22%
					TNBC: 19%
Langenhoven [26]	Cape Town, South Africa	586	55%	56	ER: 64%
					PR: 51%
					HER2: 36%
					TNBC: 16%
Amir [23]	Tanzania	937		44.7	
Rais [27]	Morocco	980	33%	46	TNBC: 16.5%

### Receptor status

Breast cancers diagnosed among African women reportedly include a disproportionate number of poor prognosis tumors, including hormone receptor negative, and triple negative. Tumors tend to be larger, with most being >2 cm. Many of the tumors are hormone receptor negative, with reported rates of both estrogen receptor (ER) and progesterone receptor (PR) negativity ranging from 36 to 79% and 30–87% [Table 1]. Few studies report on human epidermal growth factor receptor 2 (HER 2) status as its impact is over shadowed by the high rate of triple negative cancers (TNBC). Over expression of HER 2 is reported in 18% of Malian patients, 26% in South Africa, 22% in Uganda, 17.5% in Sudan, and 27% in Egypt showing differences within the continent [29–33]. Luyeye et al. compared to breast cancer molecular subtypes between Congo and Belgium and found higher Her2 over expression rates in older Congolese women compared to Belgians [34].

Many African women are diagnosed with triple negative tumors [6, 7, 23–28]. Triple-negative breast cancer (TNBC) subtypes account for 12–20% of all breast cancer; however, women of African descent tend to have a high incidence of TNBC translating into poorer outcomes [35]. The proportion of triple-negative breast cancers among all breast cancers is 23 and 28% in Tunisia and Egypt respectively [13, 27].

In summary, there is a lower average age of breast cancer diagnosis and higher stage at presentation in Africa compared to high-income countries. There is a higher proportion of triple-negative breast cancers in Africa compared to other high-income countries.

## Surgical management

Surgery is the primary modality in the management of resectable breast cancer, and when integrated with other therapies plays a significant role in controlling locally advanced or metastatic disease. However, in certain parts of the world including Africa, surgery may be the only treatment option due to limited resources for complimentary adjuvant therapies. The rates of surgical treatment vary across Africa, ranging from 35.2% in Nigeria to 100% in Cameroon; with the majority of countries reporting surgical rates between 48 and 75% compared to over 90% in European countries [26]. The differences in surgical rates could be a result of the high burden of African women presenting with unresectable breast cancer.

### Factors influencing the choice of surgery

The majority of women in developed countries present with early stage disease amenable to breast conserving techniques because of established screening and awareness programs. On the other hand, many women in Africa require radical mastectomy to control their disease [21]. On average, 50–75% of women present with very advanced disease in Africa [36]. Islami et al. reported 74 and 81% advanced stage at presentation in Cote d’Ivoire and the Democratic Republic of Congo respectively [37], Soliman et al. reported 90% of breast cancer patients present with advanced disease in Niger; invariably mastectomy is the most common surgical procedure performed [38]. Reports from Eritrea and Tanzania indicate that up to 99% of patients undergo mastectomy for various reasons including advanced stage and lack of other modalities of treatment [39, 40]. Mastectomy and breast conservation rates in Europe are reported as 30 and 70% respectively, which is in sharp contrast to 85% mastectomy rates in Africa [41]. North America reports rising breast conservation rate (68%) as at 2007 [42].

The indication for breast conserving surgery is limited early resectable disease and dependent on the availability of radiation therapy to sterilize the remaining breast tissue. Borderline resectable tumors can be down staged to allow for breast conservation with neoadjuvant chemotherapy. The poor access to radiation facilities in Africa is a major factor contributing to the limited access to conserving breast surgery in many countries. Even where radiotherapy facilities are available in Africa, very few women are considered candidates for breast conservation despite achieving good response rates to neoadjuvant chemotherapy for various reasons [5]. Maalej et al. reported that even though half of breast cancer patients present with resectable disease in Tunisia, the breast conservation rate was only 17.6% and was dependent on the surgeon’s preferences [43].

Egyptian women with early stage disease may be considered poor candidates for breast conservation because of high illiteracy rate and compounding cultural influences. These factors do not allow for regular surveillance of patients following breast conservation required to detect early recurrence in the remaining breast tissue [44].

In a recent review of breast cancer surgery in Africa, Malawi, Ghana, Rwanda and South Africa reported higher rates of lumpectomy (45, 40, 29 and 12% respectively) compared to single digit figures found in other countries [26]. This report may indicate improvement in down staging of tumors, access to care and breast cancer education. Malawi and Rwanda do not have radiotherapy facilities, meaning patients will have to travel to neighboring countries for radiotherapy and would be of interest to know the follow-up data for these patients.

### Quality of surgery

Several authors [45, 46] have discussed the inadequacy of surgical capacities to tackle cancer surgery in LMIC. Resources including skilled personnel are limited to main cities limiting access to the rural poor even though some of the required procedures are basic and could be performed by general surgeons [25]. In a few African countries like Malawi by surgeries are performed by non-physicians, who may not understand the principles underlying the adequacy of axillary dissections, obtaining clear margins and proper fixation of surgical specimens for histological assessment [47]. Economic and political instability on the continent may be a contributing factor to the limited number of trained surgical oncologist by promoting brain drain resulting from not only poor remuneration but also suboptimal resources in the working environment [46]. The continent needs to invest in the training of oncology specialist to improve outcomes [48].

### Factors contributing to poor compliance to surgery

Breast cancer patients in Africa default mastectomy for many reasons which include the fear of mastectomy. The low surgical utilization rates found in some countries could be explained by the lack of awareness in detecting early stage disease, long surgical waiting list leading to the productive use of alternative therapies, a complexity of navigating health care systems, financial constraints and illiteracy [31, 49–52].

Breast cancer surgery is considered demeaning and culturally and spiritually unacceptable [53]. The rate of mastectomy refusal varies within the continent, an example being fewer refusal rates in Eritrea and Cameroon compared to Nigeria [26].

In summary, the quality of breast cancer surgery in Africa is improving but requires an injection of resources starting with a training of surgeons with oncology skills, improving access to care and patient education on the impact of sociocultural myths.

## Systemic therapies

The number of publications documenting and detailing experiences with systemic therapies for breast cancer management in Africa continues to rise in recent years but still considered scare. For this reason, comprehensive comparisons of systemic therapy logistics and outcomes within Africa and the rest of the world was difficult in the absence of rich data.

### Chemotherapy

#### Cost and access to drugs

The cost of newer and more effective systemic therapies for cancer continues to rise. For most of Africa, meager health expenditure budgets, competing for interest and dwindling donor financial support are hindrances to accessing life-saving cancer medication with many low-middle income countries(LMIC) barely satisfying the WHO essential drug list for cancer [54]. Publications from Africa repeatedly demonstrate the limited availability of some core and newer cancer drugs and the unbearable out of pocket payments leading to treatment non –compliance [5, 36, 40]. Breast cancer activist has had little success improving access to drugs by lobbying to reduce a cost of drugs through tax exemptions and ensuring breast cancer screening, diagnosis and treatment are included in national health insurance schemes.

Breast cancer in Africa is characterized by disease in younger women with aggressive disease and poor hormone receptor staining which require the use of second and third generation drugs including Taxane-based chemotherapy [55–58]. These new drugs are considerably expensive with limited access in very few Sub-Saharan African health institutions [59–61]. Patients who are refractory to initial chemotherapy have limited options for subsequent therapies, which are either unavailable or unaffordable. Southern and northern parts of Africa have better access to newer cancer medications and promote the use of national guidelines in an attempt to standardize management of breast cancer in their countries [62].

#### Choice of drugs

Most low- and middle-income countries in Africa experience severe limitations with drug access, which unfortunately could foster the influx of cheap, suboptimal or fake drugs. Generic brands of systemic therapies have improved access to life-saving cancer medications with the promise of very low pricing and availability [63].

For each patient, the financial limitations, inconvenient scheduling of cycles, i.e., weekly versus three weekly, and a high cost of supportive therapies needed to reduce toxicity and patient and family beliefs and interferences influences the choice and sequence of treatments [64]. A pilot survey of breast cancer management in sub-Saharan Africa reports that the use of neoadjuvant chemotherapy was more prevalent and could be a result of the high burden of locally reported and or large theater waiting time. In this study cyclophosphamide, adriamycin and five Fluorouracil combination were the commonest protocol prescribed in the neoadjuvant and adjuvant setting [65]. Achieving complete pathological response rates is associated with improved survival in patients who received neoadjuvant chemotherapy. McFarland, et al. in a recent publication, describes improvements in breast cancer pathological response rates over a five-year period following neoadjuvant chemotherapy; from 14 to 43%, with an overall complete response rate of 26.5% [66]. The introduction of newer drugs into neoadjuvant protocols especially Her 2 targeted drugs, Taxanes, and carboplatin for Her2 positive and TNBC subtypes are associated with the recent improvements in pathological response rates. Sule at al., in 2016 published data on 20 patients in Nigeria who received at least five cycles of Taxane- based neoadjuvant chemotherapy, and reported a 67% complete pathological response rate [67]. Other small studies from Africa report complete pathological response rates of 10–35%, none of the studies included Taxanes, carboplatin or Her 2 targets [68–70]. These rates compare favorably with full pathological rates from developed countries before the introduction of Taxanes, carboplatin and targeted therapies [71].

A phase 2 trial conducted in Nigerian patients reported no complete pathological response rate with single neoadjuvant agent capecitabine, but documented a 44% overall response rate [72].

Breast cancer patients in Africa are known to abscond following complete clinical response to neoadjuvant, and this may influence the high mastectomy rate found in some parts of Africa [73].

## Down staging compliance to chemotherapy

In Cameroon, one-third of breast cancer patients delayed the first two cycles chemotherapy at least by 2 weeks and two third cited financial constraints as a compounding factor [74]. The cost was a major factor contributing to high noncompliance rates in other countries [39, 75]. Other than the high cost of chemotherapy, side effects of chemotherapy such as hair loss, nail changes, nausea, vomiting, and infertility is considered culturally unacceptable, driving patients to abscond treatment for traditional and less invasive unorthodox treatments [44, 76].

### Quality of service delivery

The lack of support services to manage toxicities may be a reason to withhold effective chemotherapy protocols even where accessible. In some parts of Africa, the choice and sequencing of chemotherapy protocols is ad hoc. The prescription and administration of systemic therapies are by general physicians, surgeons, and non-oncology nurses, and this negatively impacts optimization of disease control [45]. Low white cell counts levels in Africans may confound the ability to administer full doses of chemotherapy and the use highly myelo-suppressive protocols especially in non-experienced hands, notwithstanding the prohibitive cost of granulocyte stimulating growth factors [77]. The role of specialized oncology nurses is important for cancer control through education, counseling, proper administration of chemotherapy and palliation. Structured oncology nursing training programs are a necessity for Africa and other LMIC to improve the quality of cancer care [78].

## Targeted therapy

### Cost and access to drug

Epidermal Growth Factor Receptors, Vascular Endothelial Growth Factor receptor, Mammalian Target Of Rapamycin inhibitors, cyclin dependent kinases, programmed cell death protein one inhibitor either with single or double blockade of receptors targets are some of the new drugs developed to target the breast cancer cell [79]. The discovery of HER 2 targeted therapy has revolutionized the management of breast cancer. However, the cost of these life-saving drugs remains, unfortunately, astronomical even for high-income countries [80]. In 2015, Trastuzumab was included in the World Health Organisation (WHO) essential drug list for the management of Her2 positive breast cancer. However, the cost-effectiveness of this treatment in most LMIC is under debate [81]. In South and North of Africa where Trastuzumab, a HER 2 targeted drug is easily available, there are serious concerns about access [82].

Biosimilars at markedly reduced cost available to countries like India should be made available to Africa to save precious lives. The astronomical cost of biomarker testing and molecularly targeted drugs brings into question the cost-effectiveness of promoting extensive molecular profiling of breast cancer in Africa other than for research purposes.

## Hormonal therapy

This class of drugs targets the hormone receptor within the breast cancer cell. They are indicated only in hormone receptor positive disease, achieving high response rates, which translate into improved control and survival in the curative or palliative setting. The accuracy of receptor testing is dependent on efficient and reliable pathology services. Countries without these facilities resort to a blind prescription of hormonal therapies.

### Cost and access to drug

Hormonal therapy for breast cancer is one of the most available treatment options even in poorer countries. Many companies supply generic Tamoxifen at a very low cost making it readily available and in some countries available free of charge [83, 84].

Unlike Tamoxifen, the access and availability of aromatase inhibitors is restricted in most of Africa [22, 85].

### Choice of drug

In Africa, patients are more likely to be ER negative, rendering Tamoxifen ineffective in disease control if prescribed for all breast cancer patients [86, 87]. A recent meta-analysis of hormone receptor status of breast cancer patients in Africa indicates that more than half of African women have hormone positive breast cancer, disputing the poor receptor status in the majority of breast cancer cases [85]. However, this finding from the study is debatable and is not evident in clinical practice and will, therefore, require further expanded research. There are detrimental effects of prescribing Tamoxifen for negative receptor disease, and it remains apparent that improving the quality of pathology services is a key to improving survival [10]. Numerous studies have demonstrated improved outcomes with the use of aromatase inhibitors in both premenopausal (following ovarian suppression) and post-menopausal women with receptor positive breast cancer [88, 89]. The acceptance of ovarian suppression in premenopausal young African women is low and a hindrance to prescribing aromatase inhibitors as primary or second line therapy for hormone receptor positive disease. Many African countries currently recognize the importance of receptor testing and are working towards improving pathology services [90]. As an example, Madagascar did not have access to hormone receptor testing until 2011 and are currently reporting a higher ER negative- rates compared to ER-positive disease, defining a better application of targeted therapy [91].

### Compliance to hormone therapy

Non-adherence to hormone treatment is a common worldwide problem and ranges 30–72% for adherence and discontinuation [92].

Small studies from Nigeria and South Africa report 25 and 36% non-adherence rates for Tamoxifen which is comparable to data from developed countries [93, 94].

In summary, the use of systemic therapies to control breast cancer in Africa continues to improve as countries develop and adopt guidelines from developed countries. Sadly, these improvements are evident mainly in the middle and higher income countries in the continent. The impact on outcomes are be dampened by the lack of access to quality medications, unskilled personnel and socio-cultural influences in many countries.

## Radiotherapy

### Access to radiation therapy

The most challenging aspect of providing breast cancer radiotherapy in Africa is access to radiation therapy resources. More than 90% of all radiotherapy equipment is found in South and northern Africa [95]. Twenty-nine countries in Africa have no access to radiation services, and even those with these services face prohibitive maintenance costs and demands for limited skills. There is often extreme pressure on these limited resources, leading to delay in commencement of treatment. With the majority of patients being diagnosed with a locally advanced disease, the inclusion of radiation treatments in the overall management is paramount.

Expanding radiation facilities is a major step to improving outcomes in breast cancer patients with both primary and metastatic disease. Advanced techniques such as conformal and planning techniques used in HIC has been successfully replicated with less sophisticated but modernized Cobalt-60 teletherapy equipment and low energy linear accelerators as a pilot in some countries and has the potential to reduce toxicities associated with breast irradiation [96, 97]. Through partnerships with the International Atomic Energy Agency (IAEA) and other collaborators, many countries are establishing new centers or upgrading existing ones with modern radiotherapy equipment and training of technical staff, which will invariably improve access to radiotherapy services.

### Dose prescription

Treatment protocols for curative breast cancer vary across the continent with some institutions following international standardized protocols of 5 weeks of daily radiation; and in others, shortened hypo-fractionated regimes are adopted in increased throughput of patients on the limited numbers of linear accelerators [98, 99]. Also, hypofractionated regimens are unnecessarily avoided in some instances where access is only to cobalt-60 or low energy linear accelerators due to concerns regarding skin toxicities.

Radiotherapy is a cornerstone of effective palliative care, essential for managing bone pain and unresectable locally advanced disease complicated by ulceration and bleeding. A survey of patterns of palliative radiation has shown that oncologists in Africa conform to cost-effective single fractions in bone metastases but are more likely to use longer fractionation schedules for local disease [100].

### Factors affecting compliance to treatment

Interruption of radiotherapy treatments are a frequent occurrence as the daily costs of traveling for therapy can be significant [101]. Many centers in Africa charge user fees, which for the impoverished lead to a high rate of non-compliance. Data on outcomes the following radiotherapy for breast cancer patients in Africa is lacking, with no particular research being published in the last decade detailing efficacy data, abandonment or cost of treatment.

In summary, the limited radiotherapy access in Africa is a major setback to improving breast cancer outcomes considering the high burden of advanced disease on the continent.

## Managing HIV positive breast cancer in Africa

### Chemotherapy toxicities

Although the cluster of differentiation 4 (CD4) count in HIV-infected patients is not associated with age and stage of breast cancer, CD4 count at diagnosis may affect chemotherapy tolerance [102]. Langenhoven et al., in a South African cohort, reported that more than 84% of breast cancer patients, including 19 who were HIV-infected, who initiated systemic chemotherapy completed it without severe toxicity, regardless of their HIV status [26]. This report was found despite a mean decline in CD4 count during chemotherapy from 477 cells/μL to 333 cells/μL. There was no statistically significant difference in hematologic toxicity requiring dose modification. However, grade 3 or 4 lymphocytopenia developed only in the HIV-infected patients (26.4%; *p* = 0.001). Additionally, there was no data on the HIV-infected patients receiving antiretroviral therapy (ART) concurrently with chemotherapy, with scant details of the ART regimen [26].

These findings suggest that while HIV/AIDS may cause some chemotoxicity in patients with a low CD4 count, a normal CD4 count does not reduce chemotherapy tolerance. Low CD4 count, may have reduced treatment efficacy and treatment adequacy due to poor adherence, dose adjustments, treatment delays and early discontinuation of therapy [103–105].

There are no studies examining treatment outcomes with surgery and radiation therapy in patients with HIV/AIDS and breast cancer in Africa.

In summary, HIV positive breast cancer patients in Africa should be managed like HIV negative patients under proper supervision by skilled clinicians, paying attention to supportive care needs.

### Outcomes

Compared with data on the incidence and overall burden of the disease, there is a significant paucity of data on breast cancer outcomes including overall survival, quality of life and survivorship issues. Large population–based outcome reports are lacking due to various factors such as delay in diagnosis, lack or interruptions of treatment, heterogeneity and difficulty of access to screening, diagnosis and treatment and lack of high-quality population-based cancer registries [106]. Currently available data point to a very high mortality/incidence ratio of 0.55 in Central Africa as compared to 0.16 in the United States [107]. Most publications on Breast cancer treatments and outcomes are from the facility- or hospital-based case series [61]. Nonetheless, these case series provide valuable data. For example, adjuvant, chemotherapy and radiotherapy were given to 44.8 and 11.7% of patients with an overall 5-year survival of 21.8% at the Bugando Medical Center in Tanzania [40]. A retrospective study of 152 patients with triple negative breast cancer in Morocco reported with a 5-year overall survival of 76.5%. The outcome was by literature data from North America and Spain, especially in young age at high-grade diagnosis tumors, advanced stage at diagnosis, and a short time to relapse [20, 27]. Despite high response rate to chemotherapy, the overall prognosis of this subset of tumors remains poor. A recent publication from Accra, Ghana reports a 5-year overall survival of 91.94% for stage one, 15.09% for stage four and cumulative 5-year overall survival of 47.9% [108].

Increasingly reports and analysis such as treatment rates, rates of treatment adherence, local recurrence and presentation at late stages thought to be of importance in resource constraint regions are being published [40, 100].

Treatment outcomes generated from prospective clinical trials are scarce. Recent efforts to rectify this situation include the study of neoadjuvant Capecitabine chemotherapy in newly diagnosed women with advanced breast cancer in Nigeria [72]. This phase II study indicated that conducting high-quality prospective trials are feasible in resource-constrained settings and highlighted the challenges associated with generating high-quality patient outcome data such as slow accrual leading to early closure.

Poorly kept medical records and losses to follow-up of patients hamper data collection on outcomes. The African Breast Cancer-Disparities in Outcomes (ABC-DO) study is a prospective hospital-based study of overall survival, quality of life (QoL), delays in diagnosis and treatment in five African countries [109]. This study, which is underway utilizes mobile devices to capture the data of 2000 women over 3 years and will overcome traditional barriers to collecting patient-outcome data by harnessing the recent explosion of telecommunication in the continent.

In summary, reported outcomes for breast cancer interventions are scarce but show a positive trend. The poor patient follow-up culture is a major contributing factor to reporting. With improvements in local and collaborative research skills development in Africa, there should be improvements in outcomes data over the next decade.

## Future research

Although there have been significant local and global collaborative efforts to address research needs of breast cancer in Africa, critical research gaps remain in basic, translational, clinical and health services research. Integration of genomic medicine research findings in breast cancer prevention, screening, diagnosis, and treatment is significantly lagging behind in Africa [110]. In addition to research into the different and complex tumor biology of breast cancer in Africa [111], other research priorities including response to treatment, developing validated markers for chemosensitivity and radiosensitivity to guide treatment, understanding the optimal duration, sequencing and logical combinations of treatment for improved personal therapy. LMICs are societies in transition, therefore research in lifestyle changes such as diet and weight [112], hormonal influences and interaction with other non- communicable diseases(NCD) can shed light on the epidemiology of breast cancer in Africa and elucidate any changing trends in the biology of the disease. Other priorities include psychosocial and cultural dimensions, outcomes and survivorship. Traditionally personalized therapy is thought to be of more importance in high-income settings. However, given the cost of access to diagnosis and treatment in resource-limited settings, it is prudent to invest in research informing individualized, outcome-based approach to diagnosis and treatment to maximize rational use of limited resources.

Infrastructural investment enablers for research in breast cancer in Africa will require strategic planning to integrate research in cancer control plans of the continent as a whole in partnership with stakeholders including the international community. Also needed is the investment in capacity building, training of researchers and health professionals in addition to the creation of innovative programs to encourage collaborative cross-disciplinary working practices [113].

## Summary

The situation in Africa continues to show a slow progression of improved outcomes for breast cancer patients compared with the rest of the developing world. Possible reasons may include the inadequacy of health care infrastructure in many countries, poverty, limited expenditure of health budget on cancer, increasing breast cancer burden with late diagnosis, lack of continued education and awareness programs. Pathology in Africa needs to improve faster than at the present speed and the number of those who have the skills and knowledge to decipher the diagnosis becomes an urgent issue to address.

However, the outlook on some fronts calls for optimism. Many African countries are now working together to create national and international alliances to improve cancer care and therefore breast cancer care should also benefit from a more systematic approach. Increased awareness and education associated with efficient models to facilitate down staging of the disease remain essential in Africa.

## Conclusion

As more and more governments and organizations make finalizing cancer control plans a priority, guidelines and policies for breast cancer care on the continent will continue to improve. The outlook for optimism will keep on increasing as well as the survival and the quality of life for those affected by the disease. Forming consortiums should be fostered, whereby better-resourced regions in Africa could serve as mentors, striving to improve breast cancer survival on the continent.

## Abbreviations

ABC-DO: African Breast Cancer-Disparities in Outcomes
ART: Antiretroviral therapy
BMI: Body mass index
CD 4: Cluster of differentiation 4 is a glycoprotein found on lymphocytes
ER: Estrogen receptor
GLOBOCAN: Global burden of cancer study
Her 2: Human epidermal growth factor receptor 2
HIC: High-income countries
HIV: Human immunodeficiency virus
IAEA: International Atomic Energy Agency
IBC: Inflammatory breast cancer
LMIC: Low-middle income countries
NCD: Non-communicable diseases
PR: Progesterone receptor
QoL: Quality of life
SSA: sub-Saharan Africa
TNBC: Triple negative breast cancer
WHO: World Health Organization
